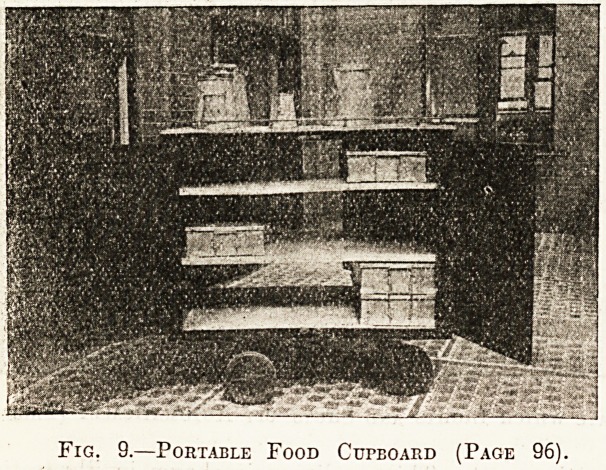# The Kitchen Equipment for a Large Hospital

**Published:** 1912-04-27

**Authors:** 


### THE MODERN INSTITUTION KITCHEN.

A carefully thought out scheme has guided the treatment of the modern institution kitchen, which is dealt with in the three special articles which follow. First, it will be noted that each writer has given a practical rather than a theoretical exposition of the subject. The following articles deal with the principles of kitchen administration as they are now being applied every day in practice at the institutions selected. For convenience of study and comparison, the subject is approached from four sides. The first article discusses the subject from the point of view of the housekeeper and the cook, and considers storage accommodation and labour-saving appliances of the most important types. The illustrations, it should be added, are of apparatus actually in use at the moment. The second article deals with "Hospital kitchens on the Continent," and here again the descriptions have been made, and the illustrations have been taken from some of the most recent apparatus employed in the famous Virchow and Beelitz-i-Mark institutions. Continental kitchens have many points to suggest to English administrators, and it is confidently believed that this will prove the most valuable summary of their kitchen arrangements that has been yet available. In so far, however, as the special hospital presents its own problems of kitchen administration, it was thought advisable to amplify the two preceding articles with one devoted to kitchen management in a special institution. For this purpose it was felt that a mental hospital would be the most instructive, for the mental hospital presents kitchen problems of an obviously unique kind. These special articles are followed by descriptions of some of the most typical of recent kitchen improvements, which thus complete the subject by presenting the hospital kitchen in detail.

### THE KITCHEN EQUIPMENT FOR A LARGE HOSPITAL.

The kitchen is one of the most important, if not the most important, administrative department of a hospital, for on the success of its arrangements and the efficiency of its staff depends the contentment of the patients and of the entire household. People, sick or well, must be fed, and unless the food is well cooked and served in an appetising manner it is not enjoyed; the distaste creates irritation of mind, and dissatisfaction soon prevails. It is essential, therefore, that a hospital kitchen be thoughtfully designed, that great consideration be bestowed upon its equipment, and that when considering the subject the point of view of the housekeeper and of the cook receive attention, as well as the question of cost of erection and equipment. It is wiser to spend money upon a large airy kitchen, with larders and pantries conveniently adjacent and with equipment which requires the minimum of labour to attain the maximum result, than to stint the initial cost of building and equipment and leave a legacy of inconvenience and discomfort to those who have to work in the department and of additional annual cost to those who have to maintain it.

In discussing this subject it is proposed, therefore, to deal with it from the aspect of the housekeeper and of the cook, to consider the question of economical labour-saving appliances, storage accommodation, distribution, the best forms of cooking apparatus, and the relative merits of electricity, gas, the open fire, and steam.

### The Housekeeper.

From the housekeeper's point of view, especially if she is also the storekeeper, it is essential that the kitchen be in such a position that it is readily accessible from her office, so that her presence on numerous occasions during the day may be looked upon as a matter of course. It is not meant by this that she should take any part in the cooking for the establishment—on the contrary, in a large institution such action on her part would be unwise; her duties should be confined to a supervision which would, while giving the cook the support of her presence at intervals, leave to that official the duty of supervising her own assistants. The housekeeper's stores for meat, milk, vegetables, bread, groceries, bacon, butter, and cheese should therefore be on a level with, and adjacent to, the kitchen, but so arranged that issues to other parts of the hospital may be made without the kitchen being invaded by porters or other servants whose duty it may be to convey milk, bread, butter, groceries, etc., to the wards and other departments of the hospital. A kitchen with the food stores and housekeeper's office arranged as indicated can be supervised by her naturally, unobtrusively, and efficiently. Adjacent to the kitchen also should be the larder for the storage of cold meat and other food which the economical housekeeper knows so well how to utilise, and it is a distinct advantage if this larder has opening from it a small refrigerating chamber for the preservation during hot weather of cooked provisions which have to be held over until the next day. The meat, milk, and butter stores should have similar cold-storage compartments, those in the larder, butter, and meat stores being maintained at about 32° Fahr. and that in the milk store at 40°.

The kitchen and its storerooms should not be on the ground floor if the building in which they are situated exceeds one storey; they should be on the top floor, and the kitchen should be fitted with an electrically-driven extraction fan, in addition to lantern-light ventilation and side and end windows. The storerooms and larders should be fitted with special air inlets just above floor level, in addition to window ventilation and extraction outlets. The tradesmen's entrance should be immediately below, and in addition to a staircase there should be an electric or hydraulic lift, so that provisions, meat, etc., can be immediately brought up for delivery at the proper store, and the tradesmen have no difficulty in reaching the housekeeper's office.

### The Cook.

It is a somewhat difficult task to arrange or equip a kitchen to the satisfaction of a cook. Cooks have their own pet theories, and arrangements made to suit one may be disliked by her successor; but there is one point upon which all cooks are agreed—namely, that the cooler a kitchen can be kept in summer the better, and this is a matter which should receive serious attention. To insure coolness it is necessary to provide a greater air space than may at first sight appear necessary, the kitchen should be spacious and lofty, the lantern lights and windows already referred to should open readily, and the electric fan be fixed in the best position for assisting ventilation. There should be a well-ventilated vegetable, fish, and washing-up kitchen or scullery adjoining, so as to keep the odours of fish and vegetables out of the kitchen proper. The walls of all stores, the kitchen and scullery should be of tiles or glazed brick, and the floors should be either of tiles or terrazzo, preferably the latter, which has an exceedingly clean appearance, and if laid in panels of comparatively small extent the danger of cracking is reduced to a minimum. It is not necessary for the cook to have access to the housekeeper's stores, but the cold-meat larder should be under her direct charge, together with its cold storage, which she will find exceedingly useful. The cold-storage

Fig. 1.—Kitchen for 800 Persons, showing Gas Ovens, Grills, Fish Fryer, and Hot-plate (Page 95).

Fig. 2.—Range of Steam Jacketed Cast-iron Boilers (Page 95.)

compartment should really be compartments, for there should be two—one for such cold meat, etc., as it may be advisable to place therein, and the other for butter and milk for kitchen use and for cooling jellies, blancmanges, etc. The cook also needs in her kitchen a large recessed store cupboard or a small storeroom in which she can keep under lock and key such of her kitchen stores as she finds necessary to be under her personal charge.

Adjoining the kitchen should be a small sitting-room, into which the cook, who should, if possible,

Fig. 3.—A Boiler Tilted (Page 95).

be a lady, can retire during the all too brief intervals of rest which she is able to snatch during her necessarily somewhat long hours of duty, and there should also be a dining-room for the kitchen staff, as it is preferable for them to have their meals apart and at different hours from the other servants, so that they may be able to partake of them in comfort and without interruption. The long series of meals which have to be despatched from a hospital kitchen would be a revelation to the chef of an hotel; and fixed hours for meals (especially dinner) for the kitchen people are a necessity if they are to be kept in health and happy and contented. The other conveniences or necessities for a well-arranged kitchen are a covered open space for the receptacles for refuse, so arranged that they can be removed without being brought through the kitchen; a food lift immediately adjacent, large enough to receive a ward food trolley and an attendant; smaller lifts descending to the service rooms attached to the dining halls of the nursing staff and servants, which should be immediately under the kitchen, also (and this may have to be outside the kitchen from the probable position of the officers' dining-room) one to the service room of the resident officers. These conveniences add considerably to the smooth working of the department.

The actual accommodation necessary for a kitchen building has now been dealt with, both from a housekeeper's and cook's point of view, and the next question for consideration is that of fittings for the various storerooms and also the kitchen apparatus.

### Storerooms.

The grocery and bread store should be equipped with a central counter, accessible at all sides and fitted with drawers with locks. On one wall there should be good wide shelving, with glass door fronts to lock (the same key should pass both drawers and

Fig. 4.—Beef-tea Infuser (Page 95.)

cupboards). One wall should be racked with skeleton shelving for bread, so arranged that one loaf cannot rest upon another. The remaining wall should provide standing room under the windows for galvanised iron bins (fitted with padlocks) for the storage of flour, rice, sugar, tea, coffee-berries, and other groceries which are bought in bulk. These bins should each be mounted on a small iron stand on wheels so that it could be wheeled without trouble to the weighing-machine, which is indispensable for the checking of supplies and for the weekly stocktaking. Each of these bins should have painted upon it the name of the article it contains and the actual weight of the bin when empty, so as to facilitate the checking of the contents at any time. An electric coffee-mill, also scales suitable for weighing all classes of groceries, etc., should be arranged upon the counter, or in such other positions as the housekeeper may find most convenient.

The vegetable store should be fitted on one side with slate or marble shelves for green vegetables, carrots, turnips, etc., the other side being without

Fig. 5.—Steamer with Four Compartments (Page 95).

shelving so as to provide suitable space for the sacks of potatoes and for a platform weighing-machine.

The milk store should be fitted with a steam chest for the sterilisation of cans; a cold-water supply, with cold-water trough for cooling them after sterilisation; a pasteurising plant capable of pasteurising, say, 200 gallons of milk per hour, as this capacity would enable supplies to be despatched to the wards rapidly and would minimise the time occupied in pasteurising and cooling. The ward milk cans should be scaled in pints to enable the proper quantities to be run direct into the cans from the cooling apparatus. Surplus milk should be kept in the cold-storage chamber.

The meat store should be fitted on one side with marble or slate shelves, and on the other, at a convenient height, with a stout flat iron bar from which to suspend the hooks for meat hanging and a steelyard for weighing carcases. A large butcher's block and the necessary tools should also be provided. The stock of meat should be kept in the cold-storage chamber during hot weather.

The butter, cheese, and bacon stores, the cook's larder, and the cold-air chambers connected therewith, should be fitted with meat bar and hooks and with suitable marble or slate slabs and shelves. The former should be equipped with a set of prints for butter pats, also a Parnell's bacon-slicing machine, which economises the consumption of ham and bacon and saves labour.

### Kitchen Fittings.

Having discussed the provision storerooms and their equipment, we next come to the kitchen, and it is necessary before deciding upon the fittings to consider carefully the respective merits of the open fire, electricity, gas, and steam.

There are many people who cling to the open fire and to a cooking-range heated by coal, contending that gas and electricity are both extravagant, and that meat roasted before an open fire or cooked in a coal range oven is more appetising than if cooked in any other way. The coal fire, however, has its drawbacks. It requires careful attention and labour in stoking if the best is to be got out of it, it is not so clean as cooking by gas or electricity, the repairs to the range in consequence of the burning out of firebricks and iron cheeks of ovens are inconvenient and far too frequent, and it is not doing the coal fire an injustice to exclude it on the grounds of the labour involved, the inconvenience of frequent repairs, and also because it is extravagant, for the consumption of coal is considerable. Between electricity and gas the chief question is one of cost of administration and maintenance, and, bearing in mind the price of electric current, the heavy consumption involved in cooking on a large scale, together with the cost of maintenance, the verdict must be in favour of gas, supplemented by steam. By their use labour is reduced to a minimum, and thus in a great institution where cooking has to be carried on for from 400 to 1,000 or 1,200 persons daily the saving of labour resolves itself into a first principle. Besides economy of labour there is a saving of cost if really high-class cooking apparatus is decided upon, for there is no economy in purchasing second or third rate gas-cooking appliances, as the cost of repairs and the extra consumption of gas will soon exhaust any saving effected in the initial expenditure.

In arranging the equipment of a large kitchen it is convenient to place the gas ovens and grills on one side, the steam-jacketed boilers and beef-tea infusers on the other, the gas hot-plate across the centre of the kitchen, and the steam hot-closet and carving and serving table towards the end nearest the exit, so that the ward food trolleys may be readily filled and despatched, and here, too, should be a 100-gallon steam kettle for boiling water, [illegible]

that a plentiful supply for filling the jackets of the dinner receptacles and for other purposes may be readily available. The steamers for potatoes, fish, etc., the fish-frying range, the steam-jacketed boiler for cooking, and the slate tank for washing green vegetables, the copper boiling vats for cleansing pots, pans, and roasting tins, the potato washer and peeler, and the machine mincer should all be in the

Fig. 6.—Two-compartment Steamer (Page 96).

adjoining vegetable, fish, and washing-up kitchen or scullery, so that all offensive odours and the noise of machinery in motion may be excluded from the main kitchen.

The appliances illustrated have been supplied to several of the largest hospitals in London and the provinces by one of the foremost firms of kitchen fitters in the country, and, although undoubtedly costly, they are most economical in administration. The range of gas ovens (illustration No. 1), the grills, the fish fryer, and the hot-plate are in constant use in an institution where cooking for 800 persons is carried out daily. The consumption of gas averages 18,500 cubic feet per week, which at 2s. 3d. per thousand amounts to £2 1s. 8d. per week, or 6s. per day for the roasting, frying, grilling, and toasting for 800 persons in a series of meals beginning at 6.30 a.m. and ending at 9 p.m.

The most suitable machine for steam-jacketed boiling coppers is a matter of opinion. Various materials have been tried for the inner lining with more or less success. Aluminium is fancied by some, and aluminium-lined steam stewing pans have been tried successfully for jam making by preserve manufacturers, but it is doubtful if this is the best material for use in a hospital kitchen, as the vegetable acids evolved in the process of cooking and the somewhat free use of soda for cleansing purposes to which the institution kitchenmaid is addicted are not conducive to prolonging the life of a steam-jacketed boiler lined with this material. Inner linings of tinned copper have been tried, but frequent retinning is necessary to keep the lining in a suitable condition for cooking purposes; copper lined with block tin has also been tried, but the expansion and contraction of the metal causes the block tin to crack; particles of food in process of cooking enter the cracks, rendering it difficult to keep the boilers sweet and clean. Boilers made of cast iron, although not beautiful in appearance, are probably the most satisfactory. There are no polishing or retinning to be done, the surface does not crack, the strongest soda solution does not injure, and, if the boiler is made without an outlet in the bottom for the purpose of drawing off the cooked liquor, there are no interstices of any sort in which particles of food can become secreted. For convenience of working this class of boiler made by the firm of engineers already referred to is by a simple device arranged to tip so that it can be readily emptied, the tipping control being so perfect that it is quite easy to pour a teaspoonful of soup and no more from one of these boilers, if such a quantity is required. Illustration No. 2 shows a range of these steam-jacketed boilers in a hospital kitchen, and No. 3 shows one tilted. An indispensable article where beef tea is preferred to Bovril, Oxo, etc., is the beef-tea infuser made by the same firm. This is also of cast iron, and it has a hot-water jacket which is kept boiling by steam, so that the beef tea in the infuser is always simmering, thus extracting the whole of the nourishment from the meat. This appliance is shown in illustration No. 4. Illustration No. 5 is a steamer divided into four small compartments, in which one cwt. or only a few pounds of potatoes may be steamed at one time with equal convenience and economy, or a variety of foods may be cooked at the same time without detriment to their flavour, or potatoes may be kept hot for a considerable time after cooking and free from discolouration by simply turning off

Fig. 7.—Hot-plate which Lights and Shuts off Automatically (Page 96).

the steam. No. 6 is a large two-compartment steamer, in which 5 cwt. of potatoes or equivalent can be cooked at one time. This steamer is also invaluable for cooking fish, pudding, ham, or any other food which requires to be cooked by moist heat. No. 7 shows a large hot-plate for frying, making sauces, or cooking generally in saucepans. An ingenious spring valve enables the gas rings to be lighted automatically by a small pilot jet when the saucepan or frying pan is put on, and the action of removing the utensil shuts off the supply of gas to the ring, which immediately goes out. This is a distinctly economical feature. No. 8 shows the gas fish-frying apparatus. It consists of three large square iron trays, 8 inches deep standing upon a gas hot-plate, each tray being fitted with a moveable wire basket, which enables the fish to be plunged into the boiling fat and lifted out when cooked.

Fig. 8.—Gas Fish-fryer (Page 96).

Fig. 9.—Portable Food Cupboard (Page 96).

A potato peeler and washer run by electric motor is one of the necessities of the vegetable kitchen; also a power mincing machine capable of chopping meat or vegetables. There are several good machines for these purposes on the market, and committees equipping hospital kitchens can easily select to meet their requirements.

A large copper vat or pair of vats for boiling greasy saucepans, roasting tins, etc., is indispensable, although rather a costly appliance, and no well-equipped kitchen should be without.

Plenty of hot-closet accommodation should be provided for keeping food warm while waiting to be despatched to the various departments, and the top of one of these hot-closets should be fitted as a carving table, especially in those institutions where it is customary for all joints to be carved in the kitchen before being despatched to the wards.

The more expensive kitchen fittings have now been discussed, but the numerous minor requirements have still to be referred to. Iron racks for saucepans must not be forgotten, and the best material for saucepans, meat dishes, etc., must also be considered. Copper saucepans look exceedingly nice, but they require much labour in cleaning and frequent retinning to keep them serviceable. It is probably preferable to select ordinary iron saucepans for the rougher work and aluminium for sauces, etc. There is a good deal of wear in the cast aluminium variety, and the expense of replacement is not now so great as it was a few years ago. Roasting dishes of sheet iron, custard or pudding dishes of tin or aluminium, pie dishes of fireproof earthenware are considered to be most serviceable; enamelled ironware is best avoided as it is apt to chip and pieces of enamel may become mixed with the food. There are, of course, numerous other articles required to equip a kitchen, an infinite variety of cans, cutlery, dishes, pots and pans, to which it is hardly necessary to refer in an article of this description. They can readily be obtained at any ironmonger's.

There is great diversity of opinion respecting the best method of despatching food to the wards. Tinned copper food receptacles, jacketed for hot water divided into compartments, with a separate lid for each compartment and with an additional lid over all, is one of the most serviceable arrangements for hot food, and a similar receptacle unjacketed for cold meat can also be recommended. Soup, beef tea, and broth require tin or aluminium cans. The former is much cheaper, and if not kept in use too long can be kept bright and clean. The best vehicle for conveying the food to the wards is a large hardwood cupboard lined with aluminium and with shelves covered with the same metal, the cupboard to be mounted on rubber-tyred wheels. Illustration No. 9 shows the portable food cupboard; the top is covered with aluminium and provided with a rail to enable the soup and beef tea to be carried outside. These retain their heat, and it is not necessary to shut them inside the cupboard during the short time occupied in transit.

## Figures and Tables

**Fig. 1. f1:**
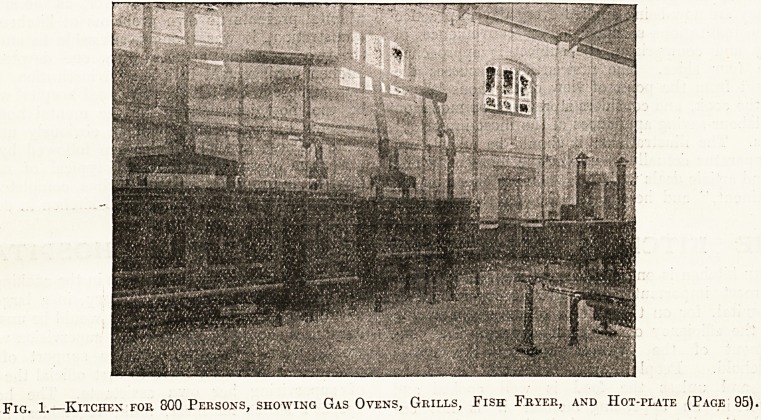


**Fig. 2. f2:**
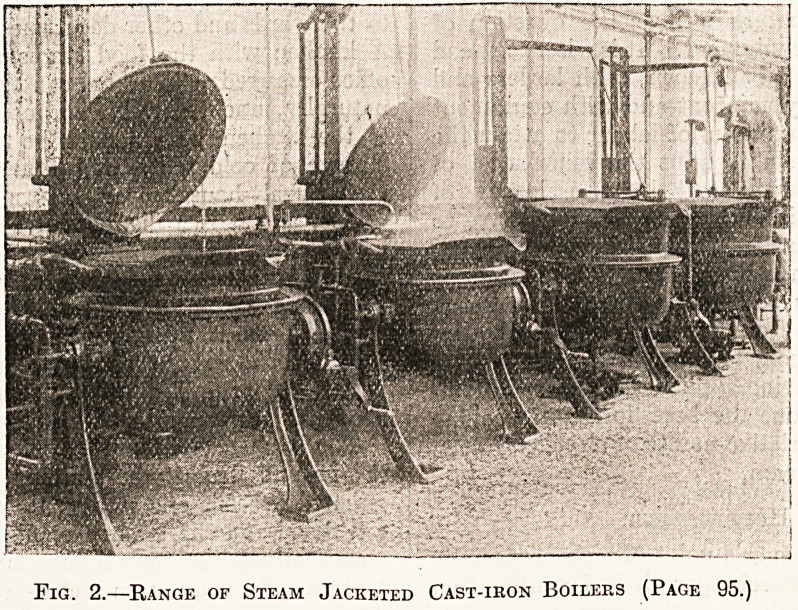


**Fig. 3. f3:**
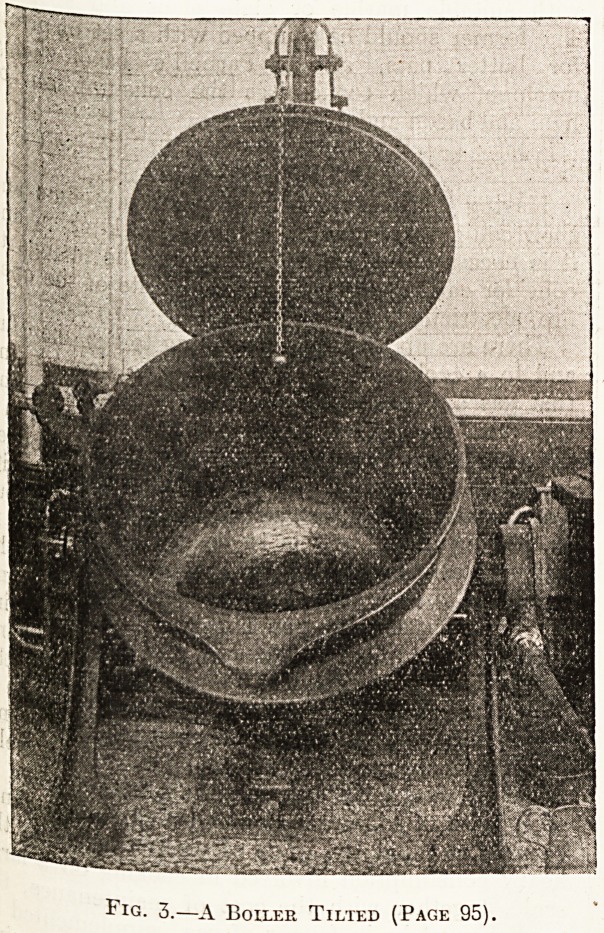


**Fig. 4. f4:**
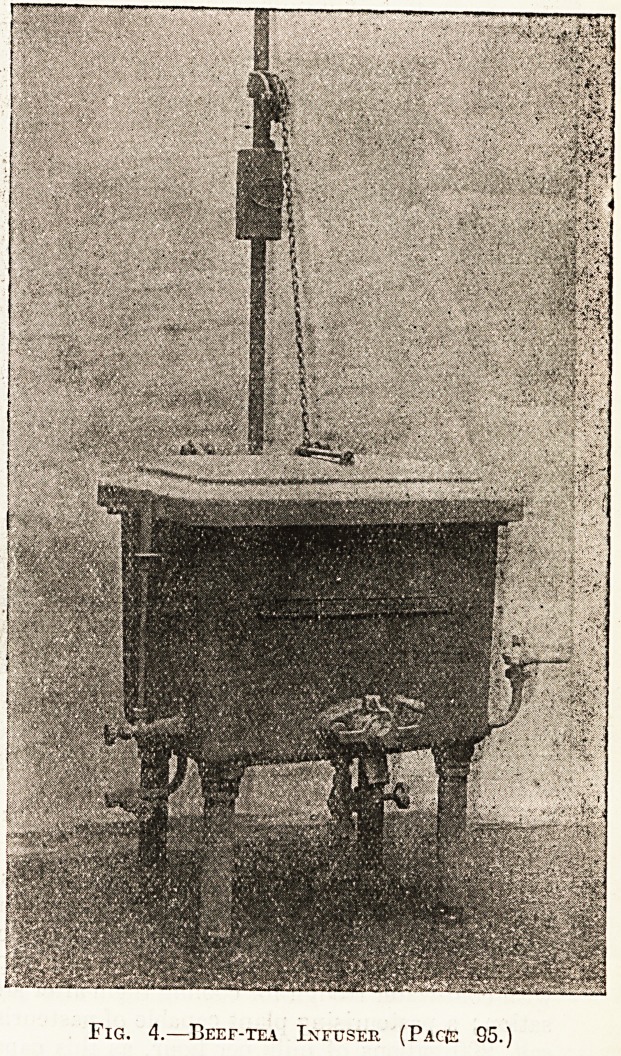


**Fig. 5. f5:**
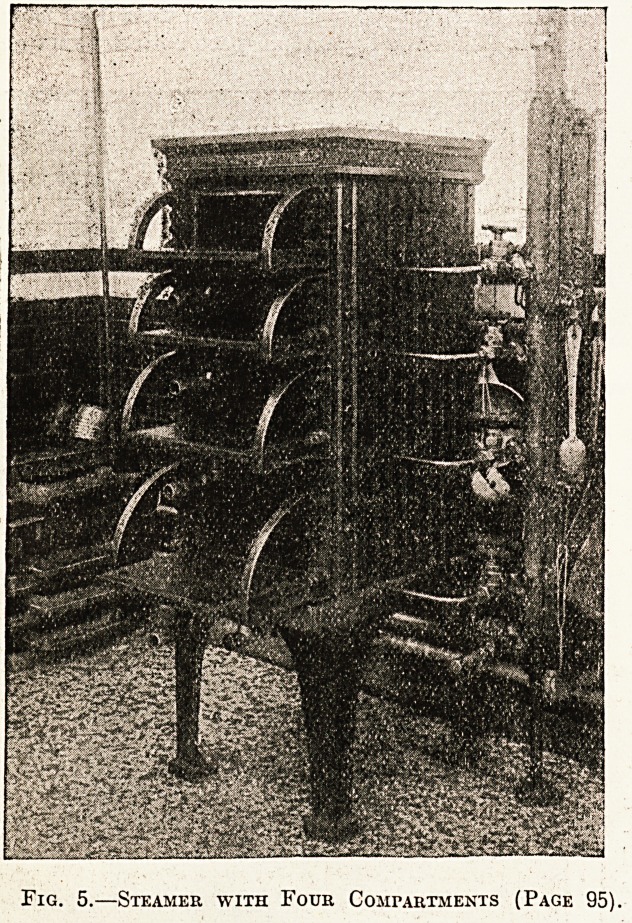


**Fig. 6. f6:**
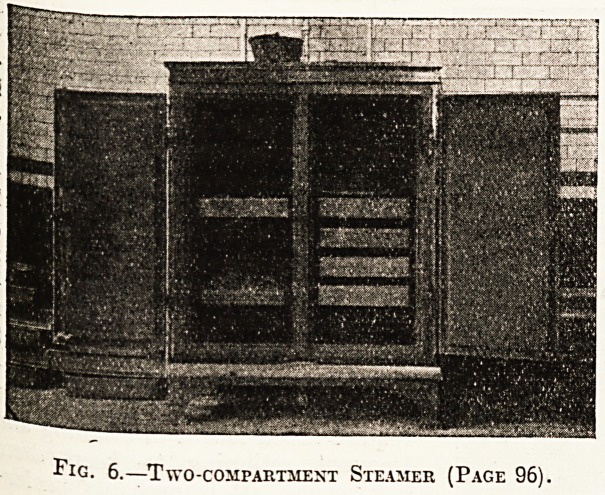


**Fig. 7. f7:**
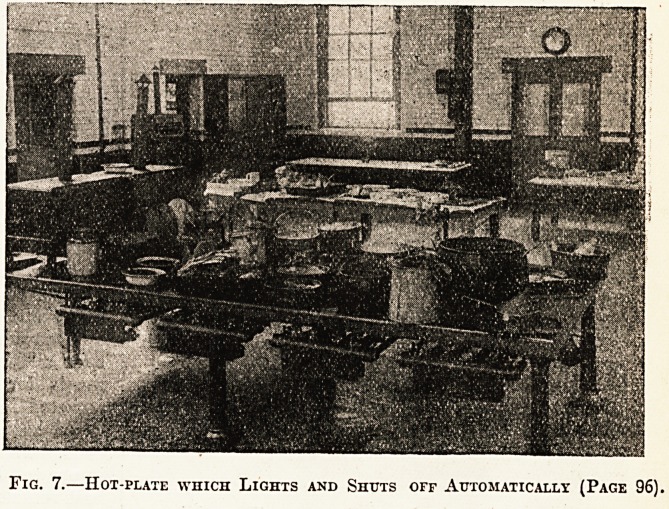


**Fig. 8. f8:**
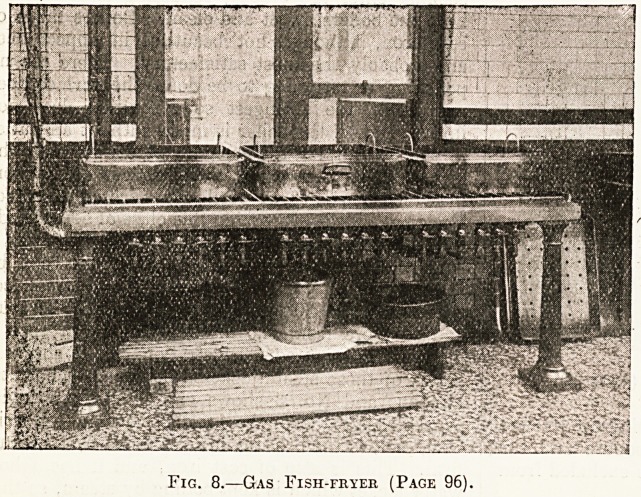


**Fig. 9. f9:**